# The Cardiac Pulsed Wave Doppler Pattern of the Common Femoral Vein in Diagnosing the Likelihood of Severe Pulmonary Hypertension: Results from a Prospective Multicentric Study

**DOI:** 10.3390/jcm13133860

**Published:** 2024-06-30

**Authors:** Marta Torres-Arrese, Pablo Barberá-Rausell, Jie-Wei Oscar Li-Zhu, Rocío Salas-Dueñas, Alma Elena Real-Martín, Arantzazu Mata-Martínez, Begoña Gonzalo-Moreno, Joaquín Hernández Núñez, Davide Luordo, Juan Gabriel Sánchez Cano, Tomás Villén Villegas, Luis Caurcel-Díaz, Gonzalo García de Casasola-Sánchez, Yale Tung-Chen

**Affiliations:** 1 Department of Emergency Medicine, Hospital Universitario Fundación de Alcorcón, Calle Budapest 1, 28922 Alcorcón, Spain; rsalasduenas@gmail.com (R.S.-D.); alma.real@salud.madrid.org (A.E.R.-M.); arantzazu.mata@salud.madrid.org (A.M.-M.); ggcasasolaster@gmail.com (G.G.d.C.-S.); 2 School of Medicine, Universidad Autónoma de Madrid, 28049 Madrid, Spain; 3 Department of Emergency Medicine, Hospital Universitario La Fe, Avenida de Fernando Abril Martorell, 106, 46126 Valencia, Spain; barbera_pab@gva.es; 4 Department of Internal Medicine, Hospital Universitario de Móstoles, Calle del Doctor Luis Montes, s/n, Móstoles, 28935 Madrid, Spain; jiewei.oscar.lizhu@hotmail.com; 5 School of Medicine, Universidad Francisco de Vitoria, M-515, Pozuelo de Alarcón, 28223 Madrid, Spain; 6 Department of Emergency Medicine, Hospital Universitario Infanta Cristina, Avenida Del Nueve de Junio, 2, Parla, 28981 Madrid, Spain; 7 Derpartment of Internal Medicine, Hospital Universitario Fundación Alcorcón, Calle Budapest 1, 28922 Alcorcón, Spain; 8 Department of Palliative Care Hospital Universitario 12 de Octubre L.CD. Av. de Córdoba, s/n, 28041 Madrid, Spain; 9 Department of Internal Medicine, Hospital Universitario La Paz. Paseo Castellana 241, 28046 Madrid, Spain; 10 School of Medicine, Universidad Alfonso X, 28691 Madrid, Spain

**Keywords:** likelihood of pulmonary hypertension, pulmonary hypertension, common femoral vein, cardiac pattern, ultrasound, emergency department

## Abstract

**Background and Objectives**: Pulmonary hypertension (PH) is a clinical condition with high mortality rates, particularly in patients over 65. Current guidelines recommend assessing the likelihood of pulmonary hypertension (LPH) using advanced echocardiography before proceeding to right heart catheterization. This study proposed using the common femoral vein (CFV), an accessible vein that reflects right atrial pressure, as an alternative method to assess the high likelihood of pulmonary hypertension (H-LPH). **Materials and Methods**: This prospective observational study included 175 emergency patients from three hospitals. Ultrasound assessed the pulsed wave Doppler (PW-Doppler) morphology of the CFV. This diagnostic yield for H-LPH was evaluated alongside traditional ultrasound parameters (right-to-left ventricular basal diameter ratio greater than 1 (RV > LV), septal flattening, right ventricular outflow acceleration time (RVOT) of less than 105 ms and/or mesosystolic notching, pulmonary artery diameter greater than the aortic root (AR) diameter or over 25 mm, early pulmonary regurgitation maximum velocity > 2.2 m/s; TAPSE/PASP less than 0.55, inferior vena cava (IVC) diameter over 21 mm with decreased inspiratory collapse, and right atrial (RA) area over 18 cm^2^). **Results:** The CFV’s PW-Doppler cardiac pattern correlated strongly with H-LPH, showing a sensitivity (Sn) of 72% and a specificity (Sp) of 96%. RA dilation and TAPSE/PASP < 0.55 also played significant diagnostic roles. **Conclusions:** The CFV’s PW-Doppler cardiac pattern is an effective indicator of H-LPH, allowing reliable exclusion of this condition when absent. This approach could simplify initial LPH evaluation in emergency settings or where echocardiographic resources are limited.

## 1. Introduction

Pulmonary hypertension affects 1% of the population and up to 10% of people over the age of 65. It is an independent mortality factor, with patients suffering from severe pulmonary hypertension having an annual mortality rate exceeding 30%, and a five-year mortality rate over 60% [1]. Unfortunately, the current definition of PH is hemodynamic, characterized by a mean pulmonary arterial pressure (PAP) over 20 mmHg at rest, measured via right heart catheterization. This test is often not feasible in everyday practice, and European guidelines recommend an initial evaluation using echocardiography to estimate the likelihood of pulmonary hypertension [2]. This algorithm classifies LPH as high, intermediate, or low based on tricuspid regurgitation velocity (TRV), and using three categories, which incorporate eight echocardiographic parameters: right-to-left ventricular basal diameter ratio greater than 1, septal flattening (left ventricular eccentricity index (LVEI) greater than 1.1 in systole and/or diastole), right ventricular outflow acceleration time less than 105 ms and/or mesosystolic notching, pulmonary artery diameter greater than the aortic root diameter or over 25 mm, early pulmonary regurgitation maximum velocity greater than 2.2 m/s, The ratio between tricuspid annular plane systolic excursion (TAPSE) and pulmonary artery systolic pressure (PASP) less than 0.55, IVC diameter over 21 mm with decreased inspiratory collapse, and right atrial area over 18 cm^2^.

The CFV is an accessible vein that can be assessed with ultrasound with a short learning curve. There are between zero and two valves between the right atrium and the CFV; thus, the pressure in the right atrium can be transmitted and assessed through the CFV. Ultrasound evaluation of patients in the emergency department can be challenging, and sometimes there is not enough experience in echocardiography, proper imaging windows, or even equipment for an adequate evaluation of the LPH. However, we face conditions such as heart failure or pulmonary thromboembolism, in which detecting significant pulmonary hypertension can be crucial and change clinical practice. This article evaluates the presence of a cardiac pattern in the CFV for detecting a H-LPH in patients presenting to the emergency department for various reasons.

## 2. Materials and Methods

A total of 175 patients who attended the emergency department for any reason and were over 18 years old were recruited. The only exclusion criterion was refusal to participate in this study. This study was approved by the local ethics committee and adhered to the principles established in the Helsinki Declaration.

**Figure 1:**
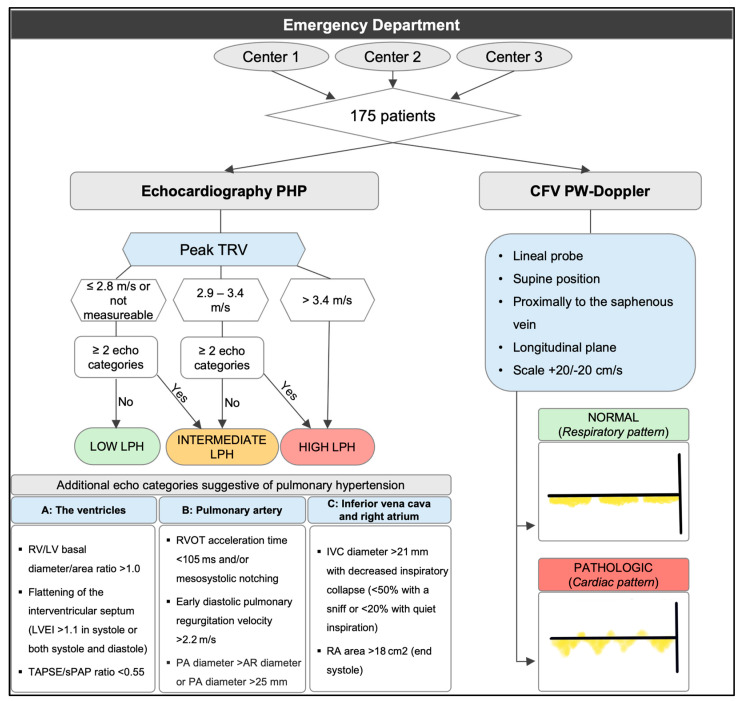
Methodology of the study. Evaluation included estimation of the LPH and assessment with PW-Doppler of the CFV. Aortic root (AR); inferior vena cava (IVC); left ventricle (LV); left ventricle eccentricity index (LVEI); pulmonary artery (PA); likelihood of pulmonary hypertension (LPH); right atrium (RA); right ventricle (RV); right ventricular outflow tract (RVOT); pulmonary artery systolic pressure (PASP); tricuspid annular plane systolic excursion (TAPSE); tricuspid regurgitation velocity (TRV).

**Figure 2:**
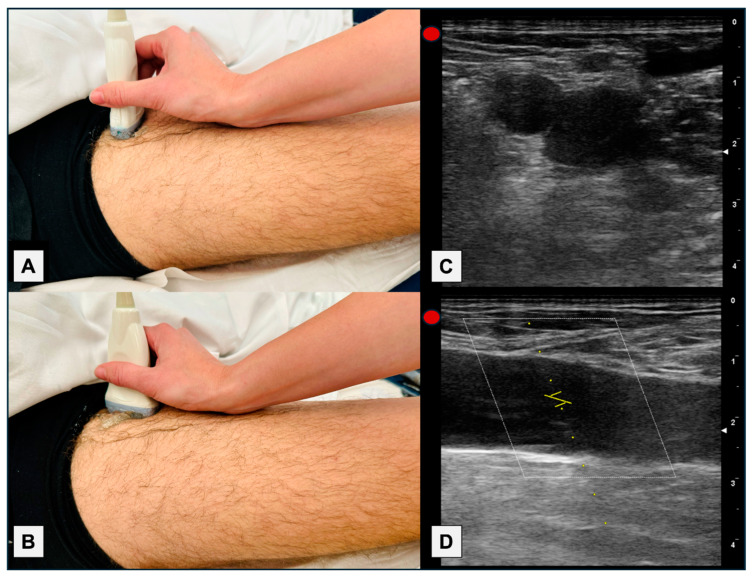
(**A**) Probe’s position on the patient for a transverse section. (**B**) Probe’s position on the patient for a longitudinal section. (**C**) Ultrasound image of the transverse section at the level of the CFV. (**D**) Ultrasound image of the longitudinal section of the right CFV. In this case, the cranial region is on the left (transducer mark) and the caudal region is on the right of the image.

This was a prospective multicentric observational study conducted in three hospitals. Due to the limited existing evidence regarding the assessment of CFV by PW-Doppler, this study was considered a pilot study, and a calculation of the necessary number of patients was not performed as such. A complete echocardiography was performed, which included all parameters mentioned for calculating LPH (Figure 1). The morphology of the PW-Doppler of the CFV was assessed using a linear probe, with the patient in a supine position (because wave morphology may vary depending on the position), proximally to the saphenous vein, after confirming the absence of deep vein thrombosis, in a longitudinal plane, and adjusting the scale to +20/−20 cm/s (Figure 1 and Figure 2). 

**Figure 3:**
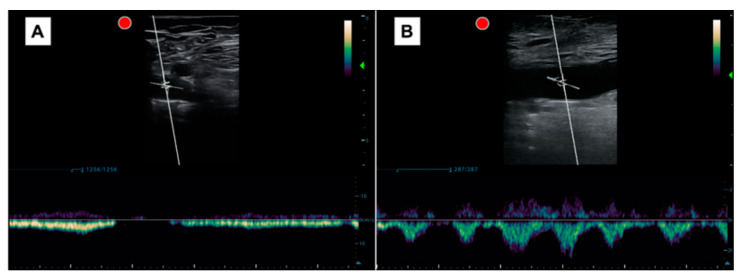
(**A**) Pulsed Doppler spectral waveform at the right CFV with a normal pattern of respiratory modulation. The flow is directed towards the heart in the expiratory phase and stops in the inspiratory phase. (**B**) Pulsed Doppler spectral waveform at the right CFV with an anormal pattern with cardiac modulation. Systolic and diastolic waves were clearly identified whether or not retrograde flow was present during spontaneous inspiration. Red dot: indicates the location of the probe marker.

The pattern obtained was interpreted as either a respiratory (normal) or cardiac (pathological) pattern. A cardiac pattern was recognized when the pulsatility was clear, and often systolic and diastolic waves were clearly identified, regardless of whether there was retrograde flow or no flow during spontaneous inspiration. When a cardiac pattern was present, spontaneous inspiration did not alter these waves, which occurs in the normal or respiratory pattern, in which waves may even disappear. A respiratory pattern, on the other hand, was defined when systolic and diastolic waves were not adequately distinguished and disappeared with inspiration (Figure 3).

A Mindray M9 (Mindray Medical Spain, Madrid, Spain) and GE VENUE (GE Healthcare, Madrid, Spain) ultrasound equipment were used. Image acquisition was performed by leading physicians with extensive experience in point-of-care ultrasound units. LPH calculation was carried out using Excel formulas to avoid errors. 

Statistical analysis was performed using SPSS 26.0 (IBM, Chicago, IL, USA). Using a Kolmogorov-Smirnov test, we verified that the variables did not follow a normal distribution. Qualitative results are expressed as percentages. Quantitative variables are expressed as mean (M) and standard deviation (SD). A comparison of the proportions of ultrasound variables was made using a Bonferroni adjustment and a significance level (*p*) of 0.05. Correlation studies were conducted using the Chi-square test, and subsequently, the performance of variables for the diagnosis of LPH was studied using the ROC curve. Sensitivity (Sn), specificity (Sp), positive predictive value (PPV), and negative predictive value (NPV) of the eight variables of the LPH algorithm and the presence of the cardiac pattern at the level of the CFV were analyzed, as well as odds ratios (OR) with their 95% confidence intervals (CI 95%) and relative risks (RR). 

## 3. Results

**Figure 4:**
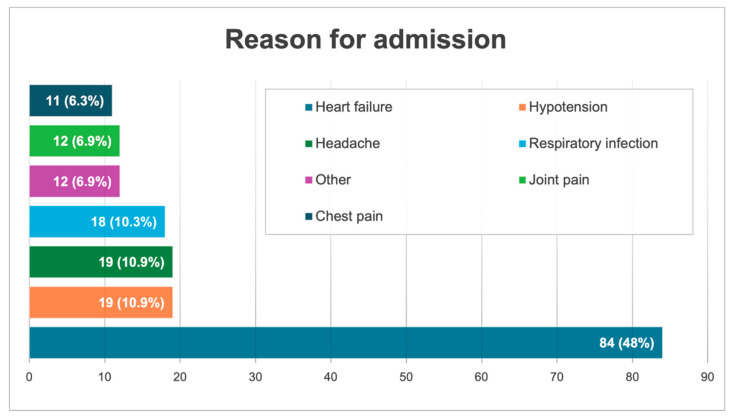
Reasons for admission. The most frequent reason for admission was acute heart failure. Other causes included pulmonary thromboembolism (*n* = 1), hypertensive crisis (*n* = 1), fever without focus (*n* = 2), or worsening of general condition (*n* = 3), among other reasons.

The subjects’ average age was 61 years (SD 20.5), and 46.3% were women. Forty-eight percent had acute heart failure, and the rest had other pathologies (Figure 4). A total of 33.1% had prior heart disease, 6% had sleep apnea-hypopnea syndrome, 12% had lung disease, and another 12% were obese. Notably, 23% had high likelihood of pulmonary hypertension (H-LPH), 34.5% had intermediate likelihood, and 61% had low likelihood. The mean pulmonary artery systolic pressure (PASP) in patients with H-LPH was 52.3 (SD 9.7), and in the intermediate–low group it was 25.1 (SD 7.3). The distribution of ultrasound parameters according to the presence of H-LPH is shown in Table 1. A Bonferroni adjustment confirmed differences in the distributions of nine ultrasound variables in patients with and without H-LPH, with a significance level below 0.05.

The correlation study using the Chi-square test showed that all parameters of the LPH algorithm were significant (Table 2), with the presence of a cardiac pattern in the CFV having the highest contingency coefficient (0.59, *p* < 0.001).

**Figure 5:**
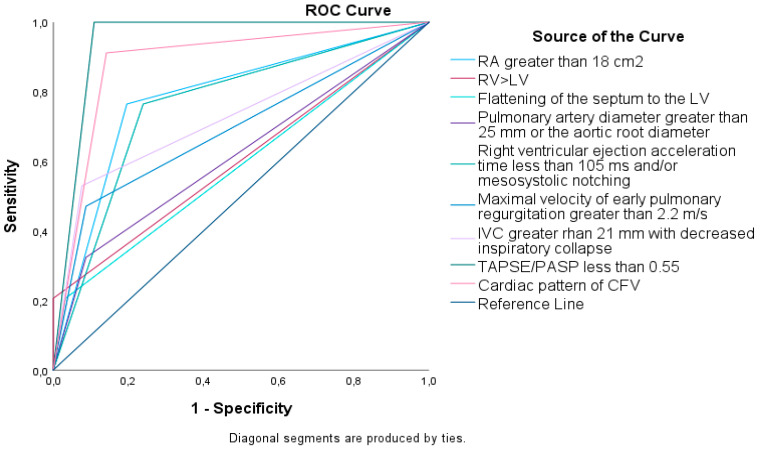
ROC curves for different ultrasound parameters in detecting H-LPH.

ROC curves (Figure 5, Table 3) indicate that parameters with the highest area under the curve (AUC) were a dilated right atrium (RA), the cardiac pattern of the CFV, and TAPSE/PASP < 0.55 (AUCs of 0.83, 0.89, and 0.96, respectively).

Sensitivity, specificity, positive predictive value, negative predictive value, odds ratios with 95% confidence intervals, and relative risks are detailed in Table 4. The presence of a femoral cardiac pattern has an RR of 18 and increases the probability of having H-LPH by 61 times (CI 95% 21–183). Additionally, it has a high specificity (96%), therefore making it less likely for the patient to have H-LPH(≤ 4%). This parameter correctly detected 72% of patients with H-LPH and had a PPV of 88% and an NPV of 90%, indicating that its absence strongly suggests the absence of H-LPH.

## 4. Discussion

The assessment of LPH is complex and may not be feasible for a physician who is not an expert in echocardiography. However, the CFV is an accessible vein, easy to scan, and holds promising potential. 

It is important to understand that between the RA and the CFV there are no connivent valves in 21% of people, one in 71% of people, two in 7%, and three in 1% [3]. Thus, whatever occurs in the RA directly transmits to the CFV. The same seems to occur in the jugular vein [4,5,6] and, with less evidence, in the subclavian vein [7,8]. Under normal conditions, during inspiration, the diaphragm moves downward, decreasing intrathoracic pressure, which generates negative pressure in the right cavities and a suction effect from the venous system. At the end of this suction effect, we even stop seeing flow in the PW-Doppler of the CFV. However, if the pressure in the right atrium increases, the suction effect disappears, and transmission of systolic and diastolic waves to the CFV occurs.

Previously, the relationship between pulsatility and pressures of the right atrium had been described by Abu-Yousef [9] and Krahenbuhl et al. [10]. The largest study conducted involved only 46 patients undergoing right catheterization, observing that the appearance of pulsatile flow is an early sign of elevated pressure in the right atrium (sensitivity of 92%) [10]. Alimoğlu et al. assessed the flow by PW-Doppler in the CFV in 30 patients with right heart failure who underwent right catheterization and observed that patients with increased pressure in the RA had a significantly higher pulsatility index [11]. Denault et al., based on descriptions of two cases, and emphasizing the direct connection between the RA and the CFV, even proposed an action algorithm in a patient with shock that subsequently has not been validated [12].

When developing this study’s protocol, we faced difficulties in choosing how to conduct the examination. The first thing we considered was what we really wanted to assess. We must consider that, being a relatively new window, there was significant variability in the variables studied at the level of the PW-Doppler flow in the CFV. In these studies, multiple parameters were evaluated: pulsatility, retrograde flow (with different speeds as cutoff points), respiratory phasicity, pulsatility index, maximum or minimum speeds, or cardiac modulation. To choose the most optimal parameter, we took into account the interesting findings of Taute et al., who assessed the presence of cardiac modulation (which is equivalent to the cardiac pattern or pulsatility without respiratory phasicity) in 47 patients with acute pulmonary embolism, and observed that all patients with a right cardiac score of ≥ 1.75 had cardiac modulation (Sn 96%, Sp 88%) [13], and our own preliminary findings, in which we observed that the pulsatile pattern in the PW-Doppler in the CFV reasonably detected elevated LPH (AUC 0.8, Sn 95%, Sp 64%, PPV 84%, and NPV 84%) in 74 patients with heart failure [14]. In our previous study, we studied the role of pulsatility, retrograde flow (which may be present if the patient has venous insufficiency), and respiratory phasicity to establish which were the best variables. Bringing together all this data, we have decided to distinguish only two patterns: the cardiac pattern, where cardiac waves are easily discernible and breathing does not result in the absence of flow; and the respiratory pattern, where waves are indistinct and breathing causes flow to disappear (Figure 3).

Another issue we considered was whether to perform measurements in the longitudinal or transverse plane. Although the transverse plane would have been simpler, and we probably would have obtained the same results (considering that the scale should be increased, as the speeds are lower), we chose to evaluate the longitudinal plane of the CFV, because all studies conducted to date used that plane, including ours. A technical issue is that when evaluated in the longitudinal plane, and with the flow perpendicular to the PW-Doppler insonation, it is necessary to perform an angle adjustment for a more adequate evaluation (Figure 3). A specific study comparing the transverse and longitudinal planes would be needed to establish their equivalence and further increase the simplicity and speed of the examination.

Other technical considerations include confirming the absence of deep venous thrombosis through compression testing and ensuring the patient is in a supine position. Positioning can influence the PW-Doppler pattern by compressing the abdomen and CFV when seated or by enhancing venous return when the legs are elevated. We must not forget to adjust the scale to +20/−20 cm/s, because if not, we might not distinguish cardiac waves.

With the previous experience gained and the methodology discussed, we decided to conduct a broader study that would increase the assessment of different pathologies in a way that closely approximates real-world environments. Although this is a pilot study, all parameters reached statistical significance, which suggests that the necessary number of patients was reached. The presence of the cardiac pattern in the CFV consolidated with the results provided as a diagnostic method for elevated LPH, regardless of the reason for consultation. Looking at results in Table 3, it is understandable that clinical practice guidelines integrate an algorithm with so many parameters to assign a likelihood. And all ultrasound variables are acceptable diagnostic tools, but none is perfect. Based on results obtained, the assessment by PW-Doppler of the CFV could be included in the algorithm for the calculation of the LPH. Certainly, in a challenging environment such as the emergency department, or in the hands of those with little experience in echocardiography, with the results of our study, we can say that the absence of a cardiac pattern at the level of the PW-Doppler of the CFV reasonably rules out the presence of significant pulmonary hypertension with a probability of 90%, and if we find a cardiac pattern, there is only a 4% chance of false positives (Sp 96%) and an 88% chance of significant pulmonary hypertension. Our results align with those previously obtained [9,10,11,13], but are probably the most relevant to date, as they cover different pathologies and involve a larger number of patients. These results turn this new diagnostic tool into a new parameter to consider in the calculation of LPH. It would be interesting to perform external validation in a different patient cohort in the future; further studies with a larger number of subjects and a higher proportion of patients with H-LPH are necessary to confirm the promising results of this form of diagnostic evaluation. 

As a limitation, as ultrasound evaluations were performed by experts in echocardiography, it may be necessary to evaluate its application by laypeople. Additionally, it should be noted that, although not applicable to real life, the gold standard would have been right catheterization; however, this limitation has been overcome by using the calculation of the likelihood of pulmonary hypertension as the target variable.

## 5. Conclusions

The cardiac pattern of the CFV is an effective indicator for detecting H-LPH, allowing this condition to be ruled out with a high degree of certainty when absent. This finding has the potential to change clinical practice in the diagnosis and management of PH in emergency settings and other areas.

## Figures and Tables

**Table 1:** Descriptive study of ultrasound variables according to the absence or presence of H-LPH.

		H-LPH	
		No	Yes
		*n* (%)	*n* (%)
RA greater than 18 cm^2^	No	115 (93.5%)	8 (6.5%)
	Yes	19 (36.5%)	33 (63.5%)
RV > LV	No	133 (80.1%)	33 (19.9%)
	Yes	1 (11.1%)	8 (88.9%)
Flattening of the septum to the LV	No	131 (79.9%)	33 (20.1%)
	Yes	3 (27.3%)	8 (72.7%)
PA diameter greater than 25 mm or the aortic root diameter	No	126 (82.4%)	27 (17.6%)
	Yes	8 (36.4%)	14 (63.6%)
Right ventricular ejection acceleration time less than 105 ms and/or mesosystolic notching	No	111 (91.7)	10 (8.3%)
	Yes	23 (42.6%)	31 (57.4%)
IVC greater than 21 mm with decreased inspiratory collapse	No	125 (86.8%)	19 (13.2%)
	Yes	9 (29%)	22 (71%)
TAPSE/PASP less than 0.55	No	122 (100%)	0 (0%)
	Yes	12 (22.6%)	41 (77.4%)
Cardiac pattern of CFV	Respiratory	120 (96%)	5 (4%)
	Cardiac	14 (28%)	36 (72%)

Right atrium (RA); right ventricle (RV); left ventricle (LV); pulmonary artery (PA); inferior vena cava (IVC); tricuspid annular plane systolic excursion (TAPSE); pulmonary artery systolic pressure (PASP); common femoral vein (CFV).

**Table 2:** Study of correlations of ultrasound variables with H-LPH.

Ultrasound Parameters	Pearson Chi-Square	Contingency Coefficient
RA greater than 18 cm^2^	66.094	0.524
RV > LV	22.663	0.339
Flattening of the septum to the LV	15.902	0.289
PA diameter greater than 25 mm or the aortic root diameter	22.677	0.339
Right ventricular ejection acceleration time less than 105 ms and/or mesosystolic notching	50.263	0.472
Maximal velocity of early pulmonary regurgitation greater than 2.2 m/s	33.271	0.4
IVC greater than 21 mm with decreased inspiratory collapse	47.46	0.462
TAPSE/PASP less than 0.55	50.57	0.473
Cardiac pattern of CFV	92.055	0.587

**Table 3:** Areas under the curve for different ultrasound parameters in detecting H-LPH.

Area under the Curve			
Test Result Variable(s)	Area	Asymptotic 95% Confidence Interval	
		Lower Bound	Upper Bound
RA greater than 18 cm^2^	0.832	0.753	0.910
RV > LV	0.594	0.487	0.701
Flattening of the septum to the LV	0.586	0.480	0.693
Pulmonary artery diameter greater than 25 mm or the aortic root diameter	0.641	0.535	0.747
Right ventricular ejection acceleration time less than 105 ms and/or mesosystolic notching	0.792	0.707	0.877
Maximal velocity of early pulmonary regurgitation greater than 2.2 m/s	0.686	0.582	0.790
IVC greater than 21 mm with decreased inspiratory collapse	0.735	0.635	0.835
TAPSE/PASP less than 0.55	0.955	0.926	0.985
Cardiac pattern of CFV	0.887	0.821	0.952

Right atrium (RA); right ventricle (RV); left ventricle (LV); inferior vena cava (IVC); tricuspid annular plane systolic excursion (TAPSE); pulmonary artery systolic pressure (PASP); common femoral vein (CFV). Asymptotic significance was less than 0.05 in all cases.

**Table 4:** Study of the diagnostic yield of ultrasound parameters for detecting H-LPH.

Ultrasound Parameters	Sig.	OR	Inferior CI 95%	Superior CI 95%	RR	Sn	Sp	PPV	NPV
RA greater than 18 cm^2^	0	25	10.027	62.165	9.76	63%	93%	80%	86%
RV > LV	0.001	32	3.895	266.879	4.47	89%	80%	20%	99%
Flattening of the septum to the LV	0.001	11	2.661	42.108	3.61	73%	80%	20%	98%
Pulmonary artery diameter greater than 25 mm or the aortic root diameter	0	8	3.118	21.393	3.61	64%	82%	34%	94%
Right ventricular ejection acceleration time less than 105 ms and/or mesosystolic notching	0	15	6.443	34.739	6.95	57%	92%	76%	83%
Maximal velocity of early pulmonary regurgitation greater than 2.2 m/s	0	11	4.352	27.15	4.29	67%	84%	44%	93%
IVC greater than 21 mm with decreased inspiratory collapse	0	16	6.451	40.09	5.38	71%	87%	54%	93%
TAPSE/PASP less than 0.55	0.9	Inf				77%	100%	100%	91%
Cardiac pattern of CFV	0	61	20.813	182.991	18	72%	96%	88%	90%

Significance (sig.); odds ratio (OR); confidence interval (CI); relative risk (RR); sensitivity (Sn); specificity (Sp); positive predictive values (PPV); negative predictive values (NPV); right atrium (RA); right ventricle (RV); left ventricle (LV); inferior vena cava (IVC); tricuspid annular plane systolic excursion (TAPSE); pulmonary artery systolic pressure (PASP); common femoral vein (CFV). Inf: infinite. A count equal to 0 in TAPSE/PASP < 0.55 complicates calculations.

## Data Availability

Data are not available due to privacy or ethical restrictions, but can be provided if desired in accordance with MDPI’s Research Data Policies at https://www.mdpi.com/ethics.
